# *Bifidobacterium animalis* ssp. *lactis* 420 Protects against Indomethacin-Induced Gastric Permeability in Rats

**DOI:** 10.1155/2012/615051

**Published:** 2012-07-17

**Authors:** Anna Lyra, Markku Saarinen, Heli Putaala, Kaisa Olli, Sampo J. Lahtinen, Arthur C. Ouwehand, Mari Madetoja, Kirsti Tiihonen

**Affiliations:** ^1^DuPont Nutrition and Health, Kantvik Active Nutrition, Sokeritehtaantie 20, 02460 Kantvik, Finland; ^2^Toxis, SBW Corp. Ltd., Lemminkäisenkatu 14-18 C, 20520 Turku, Finland

## Abstract

Gastrointestinal (GI) adverse effects such as erosion and increased permeability are common during the use of nonsteroidal anti-inflammatory drugs (NSAIDs). Our objective was to assess whether *Bifidobacterium animalis* ssp. *lactis* 420 protects against NSAID-induced GI side effects in a rat model. A total of 120 male Wistar rats were allocated into groups designated as control, NSAID, and probiotic. The NSAID and probiotic groups were challenged with indomethacin (10 mg/kg^−1^; single dose). The probiotic group was also supplemented daily with 10^10^ CFU of *B. lactis* 420 for seven days prior to the indomethacin administration. The control group rats received no indomethacin or probiotic. The permeability of the rat intestine was analysed using carbohydrate probes and the visual damage of the rat stomach mucosa was graded according to severity. *B. lactis* 420 significantly reduced the indomethacin-induced increase in stomach permeability. However, the protective effect on the visual mucosal damage was not significant. The incidence of severe NSAID-induced lesions was, nevertheless, reduced from 50% to 33% with the probiotic treatment. To conclude, the *B. lactis* 420 supplementation protected the rats from an NSAID-induced increase in stomach permeability and may reduce the formation of more serious GI mucosal damage and/or enhance the recovery rate of the stomach mucosa.

## 1. Introduction

Nonsteroidal anti-inflammatory drugs (NSAIDs) are commonly used to relieve pain and fever but also typically cause gastrointestinal side effects such as mucosal injury. The pathophysiology of NSAID-induced injuries is considered to be either a prostaglandin (PG)-dependent or non-PG dependent mechanism [1]. The PG-dependent mechanism refers to the inhibition of cyclooxygenase (COX), leading to decreased mucosal PG. Traditional nonselective NSAIDs, such as aspirin, ketoprofen, indomethacin, and diclofenac, affect the expression of COX-1 and COX-2 present in the gastrointestinal (GI) membrane [2]. The suppression of COX-2 alleviates inflammation, whereas the simultaneous suppression of COX-1 hampers the prostaglandin production essential for mucin formation and a functional epithelial barrier in the GI tract [2, 3]. Thus GI adverse effects such as erosion and increased permeability are common during the long-term use of non-selective NSAIDs [2–4]. Next-generation NSAIDs that selectively inhibit COX-2 are less prone to causing moderate GI side effects [4], although complicated side effects are as common among selective COX-2 inhibitor users as among traditional NSAID users [5, 6]. Recent studies on the pathogenesis of NSAID-induced mucosal injury indicate that NSAIDs inhibit oxidative phosphorylation in epithelial cell mitochondria, independently of the PG-dependent mechanism. The resulting mitochondrial dysfunction leads to disturbances in cellular energy metabolism and ion regulation, causing increased intestinal permeability and mucosal damage [7].

The host GI microbiota may enhance or reduce the risk of NSAID side effects. In 1996, Uejima and colleagues demonstrated a connection between 5-bromo-2-(4-fluorophenyl)-3-(4-methylsulfonylphenyl) thiophene (BFMeT)-induced ileal ulceration and intestinal microbiota in rats [8]. A decrease in Gram-positive rods and an increase in Gram-negative rods, including *Escherichia coli, Klebsiella,* and *Proteus,* was observed due to ulceration [8]. Moreover, *Lactobacillus acidophilus* ATCC4356 and *Bifidobacterium adolescentis* ATCC15703 were shown to repress ulcer formation in rats, putatively by inhibiting the growth of Gram-negative rods [8, 9]. In elderly human subjects, a decrease in lactobacilli and actinobacteria due to NSAID use has been observed [10]. Within actinobacteria, mainly the numbers of *Collinsella *[10] were reduced, which has previously been reported for functional bowel disorder sufferers [11] and colon cancer patients [12]. Thus, an intriguing alternative for protecting humans from NSAID-induced side effects is the parallel use of probiotics [13, 14]. Indeed, certain probiotic [15] strains induce epithelial cell proliferation and mucus secretion, thus potentially beneficially affecting NSAID-induced adverse effects [16], and are capable of stabilizing distorted GI microbiota [17].

To date, a limited number of studies have investigated the potential protective effect of different probiotic supplements against NSAID-induced gastrointestinal damage with varying outcome measures. *In vitro *studies with *Lactobacillus casei *DN-114 001 [18] and animal studies applying *Lactobacillus casei *strain Shirota [19] and a multistrain mixture of human origin [20] have yielded promising results. In clinical trials, *Lactobacillus rhamnosus *GG (LGG) has been shown to reduce indomethacin-induced gastric permeability [13] and the multistrain supplement VSL#3 has been shown to alleviate inflammation caused by indomethacin [14]. Moreover, *Lactobacillus acidophilus *NCFM and lactitol may protect against the GI microbiota alterations associated with NSAID use [21] among elderly subjects regularly consuming NSAIDs [22]. Taken in parallel with NSAIDs, probiotics are a promising complementary treatment for relieving NSAID-induced adverse effects. However, few studies assessing the subject have been conducted thus far, especially as regards bifidobacteria, and, as is commonly known as a characteristic of probiotics, the putative protective effect against NSAID-induced side effects is also strain-specific [19, 23].

Our objective in the present study was to analyse whether *Bifidobacterium animalis *ssp. *lactis* (*B. lactis*) 420 has a protective effect against NSAID-induced GI damage in an animal model. Since the molecular bases of NSAID-induced GI damage and the NSAID therapeutic effect are due to COX-1 and COX-2 suppression, respectively, we chose *B. lactis *420 as a candidate probiotic for our study. *B. lactis *420 has been shown to upregulate COX-1 expression and to suppress COX-2 expression in Caco-2 cells [24, 25] and to produce fermentation products capable of enhancing the epithelial barrier [25, 26]. We conducted three separate studies using a well-described rat model based on indomethacin-induced GI damage [27]. All studies included identical control, NSAID and probiotic groups. Additional arms were added to individual studies to allow the testing of the dose-responsiveness of live *B. lactis *420 cells and the effect of *B. lactis *420 metabolites. As outcome measures, we focused on both the permeability of the stomach and small intestine, and on the visual examination of gastric mucosal damage [27, 28].

## 2. Materials and Methods

### 2.1. Animals

All animal experiments were conducted at Toxis *In vivo *Services (SWB Corp. Ltd., Turku, Finland) and approved by the National Animal Care and Use Committee and performed according to the Guidance Document on the Recognition, Assessment, and Use of Clinical Signs as Humane Endpoints for Experimental Animals Used in Safety Evaluation (Environmental Health and Safety Monograph Series on Testing and Assessment no 19. OECD 2000) guidelines. In total, 160 clinically healthy male Wistar rats (HsdBrlHan:WIST, Harlan Netherlands, Horst, NL) aged between 8 to 9 weeks with an average weight of 254 g (individual weights deviated by less than 10% from the average weight per study) were acclimatised to the facility for 6 days and housed in either aspen chip-bedded cages of 2 to 3 animals or separately in metabolic cages. Raised bottom grids were used in the animal cages during the fasting period. The room temperature was kept at 21 ± 3°C and relative humidity at 55 ± 15%. Artificial lighting was applied in 12 hour shifts. The animals were provided with both nonsterile Formulab Diet 5008 and tap water *ad libitum*. Animals were clinically examined twice a day during the week and once a day during the weekends. All clinical signs were reported.

### 2.2. Chemicals

Sucrose was from Suomen Sokeri Oy (Kantvik, Finland). Sterile water was obtained from Baxter (Helsinki, Finland). Unless indicated otherwise, all other reagents were from Sigma (Sigma-Aldrich, St. Louis, MO, USA). 

### 2.3. Supplements

All supplements were given to the test animals *per os *(p.o.) using a gavage at a volume of 10 mL/kg^−1^ of weight (approximately 2.5 mL per animal) or exactly 2.5 mL per animal. All animals were observed for any abnormal signs during dosing in the morning and after dosing in the afternoon. Live *B. lactis* 420 cells (DSM 22089; Danisco, Niebüll, Germany) were administered at 10^10^ CFU·d^−1^ (high dose) or 10^8^ CFU·d^−1^(low dose) per animal. The cell-free extract was prepared by cultivating *B. lactis* 420 in Man, Rogosa, and Sharpe (MRS, Oxoid Ltd, Cambridge, UK) broth anaerobically at 37°C until OD600 was 2.0, which roughly corresponded to a bacterial cell count of 5 × 10^8^ mL^−1^ as determined by flow cytometer [29]. The bacterial cells were removed by centrifugation at 30.000 ×g for 15 min (Beckman Coulter Avanti J-20 Xpi, Brea, CA, US) and the cell-free extract was prepared by evaporating the supernatant with Rotavapor (Büchi Rotavapor R-200, Flawil, Switzerland) at +40°C to 1/13.5 of its original volume. The extract was then diluted to correspond to a bacterial density of 10^10^ CFU·mL^−1^. The L-lactic acid supplement was adjusted to the amount of acetic and lactic acid in the cell-free extract (52.4 mM).

Indomethacin was supplied at 10 mg/kg^−1^ in a 50 g·L^−1^ sodium bicarbonate solution. The carbohydrate probes; sucrose (1 g), lactulose (120 mg), and mannitol (80 mg) were given p.o. in a 2 mL volume of sterile water [27]. 10 *μ*L of 10% thymol in isopropanol was added to the urine collection tubes to prevent microbial degradation of the probes.

### 2.4. Permeability Probe Quantification

Sucrose, lactulose and mannitol in rat urine were determined by high pH anion exchange chromatography after purification of the samples by solid phase extraction (SPE). The SPE cartridges (Bond Elut SCX, 500 mg, Varian, Palo Alto, CA, USA) were preconditioned with 2 mL of methanol followed by 2 mL of water. The urine sample (0.5 mL) was passed through the SPE cartridge and the effluent was collected into a test tube. Thereafter, the analytes were eluted with 3 mL of water into the same test tube and the sample was diluted to 50 mL with water. The concentrations were determined using a Dionex HPLC system (Sunnyvale, CA, USA) equipped with ED50 pulsed electrochemical detector (PED), GP50 pump and AS50 sampler. For determination of sucrose and lactulose, a CarboPac PA-1 column (precolumn 4 × 50 mm and analytical column 4 × 250 mm) and gradient elution with a mobile phase that consisted of a mixture of (A) water, (B) 0.2 M NaOH, and (C) 0.2 M NaOH and 0.5 M sodium acetate was used. The gradient was 0–8 min, A = 84% and B = 14%; 22–30 min, A = 44 and B = 34%; 31–41 min, A = 84% and B = 14%. The flow rate was 1 mL/min^−1^ and the column temperature 35°C. A solution of 0.3 M NaOH was added to the column effluent before the PED cell at a flow rate of 0.6 mL/min. For determination of mannitol, a CarboPac MA1 column (precolumn 4 × 50 mm and analytical column 4 × 250 mm) and gradient elution with a mobile phase that consisted of a mixture of (A) water and (B) 1 M NaOH was used. The gradient was: 0–1.1 min, A = 40%; 19–27 min, A = 10%; 27.1–40 min, A = 40%. The flow rate was 0.4 mL/min^−1^ and the column temperature 35°C. The concentrations of carbohydrates in urine were calculated using the external standard method.

### 2.5. Histological Examination

Animals were euthanized by carbon dioxide before necropsy. After euthanasia, gross necropsies were performed for all animals. The total damaged area (TDA) of the intestine (mm^2^) was calculated by observing visual lesions with 40x magnification from stomach mucosa rinsed with physiological salt solution (0.9% NaCl). The observations were graded as follows; Grade 0 as normal; Grade I as mild, slight, few, or small lesions (number of lesions less than 10); Grade II as moderate in the appearance, size, or number of lesions (number of lesions 10 to 20); Grade III as severe, massive, or extensive lesions in terms of the number or size (number of lesions more than 20). Grade III represented the maximal change in the macroscopic examination.

### 2.6. Experimental Design of Animal Studies

**Figure 1:**
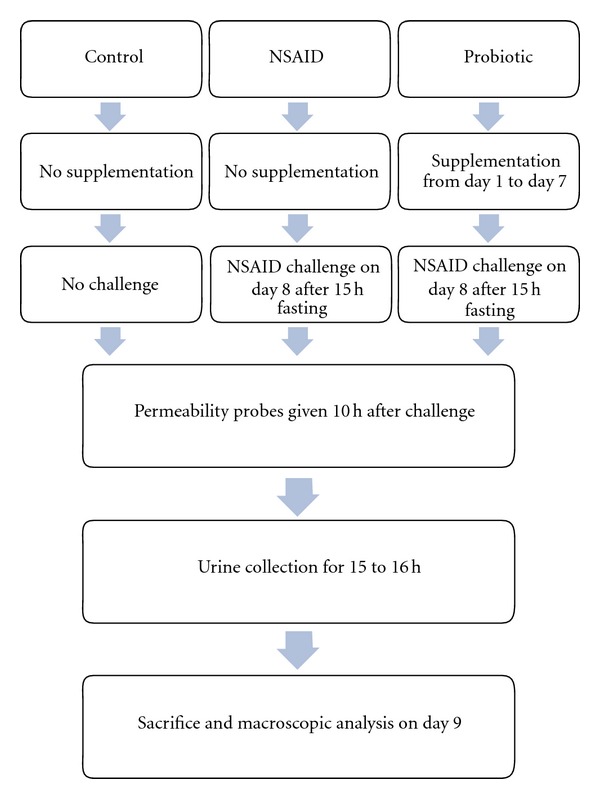
Study protocol outline followed in animal studies. The probiotic group rats were given live *Bifidobacterium lactis* B420 cells (high dose or low dose), a cell-free extract or pure lactic acid as supplementation.

Three separate studies, including a total of 160 rats, were conducted. In assessing the effect of the high probiotic dose, the results concerning the relevant treatment groups were combined from all three studies and presented as Study I. The active groups were given live bacterial cells (high dose or low dose), a cell-free extract, or lactic acid (Figure 1). The control and indomethacin groups were given sterile water as a placebo supplement. Active and placebo treatments were administered for seven days prior to the indomethacin challenge on day eight. After a 15- to 16-hour fasting period, indomethacin was given p.o. to induce GI damage. Ten hours later carbohydrate probes (sucrose, lactulose, and mannitol) were given to determine the permeability of the stomach and the small intestine, respectively [27]. Thereafter, faeces and urine were collected separately for 15 to 16 hours, using rat metabolic cages (Tecniplast, Brianza, Italy). The urine samples were stored at −20°C until analysis. On day nine, the animals were euthanized and a gross necropsy was performed to evaluate macroscopic damage in the stomach and the disaccharide concentrations were measured from the rat urine.

The setup of the separate studies is presented below.

**Study I: Protective Effect of B. lactis 420**

The protective effect of live *B. lactis *420 was tested in three trials with an identical protocol and three identical equal size treatment groups consisting of a total of 40 male Wistar rats in each group (control, indomethacin challenge, and probiotic high dose; *n* = 120). The probiotic group received the high dose of *B. lactis *B420. The results from all three trials were combined for analysis.

**Study II: Dose-Responsiveness of B. lactis 420**

For assessing the dose-responsiveness of *B. lactis *420, four treatment groups of 10 male Wistar rats each (*n* = 40) designated (1) control, (2) indomethacin challenge, (3) probiotic low dose, and (4) probiotic high dose, were analysed.

**Study III: Effectiveness of B. lactis 420 Cell-Free-Extract and Lactic Acid**

The effectiveness of *B. lactis *420 metabolites for mediating a gastroprotective effect during NSAID use was tested in comparison to live cells and pure lactic acid. Each treatment group consisted of 15 male Wistar rats (*n* = 75); (1) control, (2) indomethacin challenge, (3) probiotic high dose, (4) cell-free extract, and (5) lactic acid.

### 2.7. Statistical Analyses

All numerical data are presented as mean values with standard deviations (SDs). The gastric and small intestinal permeability were expressed as the urinal amount of sucrose and the urinal lactulose : mannitol-ratio, respectively. For TDA, analyses were also performed with values weighted according to the degree of mucosal damage (1/10, 3/10, and 6/10 for damage areas of Grades I, II, and III, resp.). One-way ANOVA with Tukey's multiple comparison test was used for permeability measurements and nonparametric Kruskal-Wallis test followed by Dunn's multiple comparison test were used for TDA. The analyses were performed with Prism 5 Version 5.01 (GraphPad Software, Inc., San Diego, USA).

## 3. Results

### 3.1. Animal Trials

In the animal trials, all except one rat remained alive during the intervention and no significant difference was detected between the weights in the different treatment groups (data not shown). The death of one rat in the trial analysing the effect of *B. lactis *420 metabolites and lactic acid was examined and found not to be due to any supplement given within the study. Hyperemia in the stomach of test animals was detected in all studies in all treatment groups including the control group receiving no indomethacin or probiotic and was therefore not analysed as an outcome measure.

### 3.2. Protective Effect of Live *B. lactis* 420

**Figure 2:**
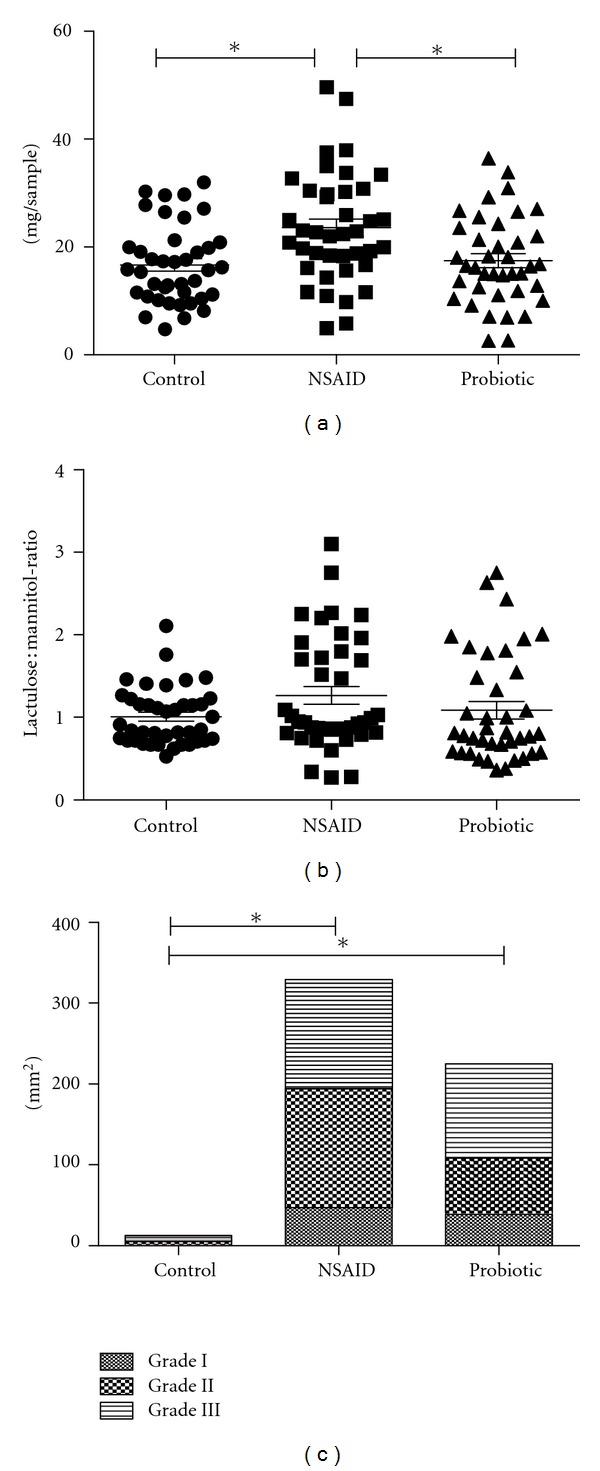
Protective effect of *Bifidobacterium lactis* 420 against indomethacin-induced side effects. Quantities of permeability probes sucrose (a) and lactulose : mannitol (b) in rat urine samples and the total damage areas (TDA) of gastric mucosa (c) combined from three individual studies (*n* = 119). The treatment groups were a control group, an indomethacin-challenged group (NSAID), and a probiotic group supplemented daily with 10^10^ colony forming units of *Bifidobacterium lactis* 420 for seven days prior to indomethacin challenge. Sucrose measures gastric permeability and the lactulose : mannitol-ratio reflects small intestinal permeability. TDA values include Grade I (mild, slight, few, or small), Grade II (moderate appearing, size, or number), and Grade III (severe, massive, or extensive in number or size) lesions. The vertical lines in figures (a) and (b) represent mean values. Significant (*P* < 0.05) differences between treatment groups are denoted by an asterisk.

In the combined analysis (Study I), indomethacin caused a significant increase in mucosal permeability and TDA (Figure 2). Sucrose was given as a marker to evaluate gastric permeability and lactulose and mannitol were given to evaluate small intestinal permeability. Gastric permeability was significantly increased in the NSAID group, whereas the small intestine remained unaffected. In the *B. lactis* 420-supplemented group (high dose), the sucrose levels remained comparable with the control group, regardless of the NSAID challenge, implying a protective effect against NSAID-induced increased gastric permeability. The lactulose : mannitol levels in the probiotic group also remained comparable with the control group.

The stomach mucosa of all rats was visually analysed for lesions which were graded according to severity. Significant damage to the stomach mucosa was detected (Figure 2(c)), whether analysed by weighted or nonweighted TDA values (Table 1). However, despite the positive direction of the effects, the protective effect of *B. lactis* 420 on the stomach mucosa was not significant according to the TDA values (Figure 2(c), Tables 1 and 2). No significant difference was seen in the distribution of the most severe type of lesions (Grade III) between the *B. lactis* 420-supplemented group and the group only challenged with indomethacin (Table 2). The mean sucrose values measured from rat urea did not correlate with the presence of lesions on the stomach mucosa, but appeared relatively stable within each treatment group (Table 3).

### 3.3. Dose-Responsiveness and Effect of Metabolites

In the individual rat studies assessing dose-responsiveness (Study II) and the effect of metabolites (Study III) of *B. lactis *420, only the gastric permeability was significantly affected by the NSAID challenge, but the TDA values remained at a low level even within the indomethacin challenge group not administered by the probiotic (Table 4). On the other hand, in the metabolite study the TDA values showed a significant increase due to indomethacin, but the permeability of neither the stomach nor the TDA values were affected (Table 5). None of the treatments (high or low dose of live *B. lactis *420, cell-free extract supplementation, or lactic acid) resulted in a statistically significant protective effect in the individual studies, Studies II and III (Tables 4 and 5). Nevertheless, these results still contributed to the positive effects of the pooled data set (Study I).

## 4. Discussion

Despite the GI-related side effects, such as ulceration and increased epithelial permeability, occasional and long-term NSAID use is common. Specific probiotic strains have anti-inflammatory effects including protection from and enhanced healing of ulceration in colitis and the capability of enhancing the GI epithelial barrier [16, 30]. Therefore, probiotics provide an intriguing alternative as a protective dietary supplement during NSAID consumption. The probiotic strain selected for the present study, *B. lactis *420, has previously been shown to affect the COX-1 and COX-2 expression profile in Caco-2 cells in a manner opposite to that of the expected effect of NSAIDs [24, 25]. Moreover, *B. lactis *420 is capable of enhancing the intestinal epithelial cell barrier in a Caco-2 cell monolayer [25, 26].

In the current study, identically treated groups from three independent studies were combined (3 treatment groups, *n* = 119) in order to assess the protective effect of *B. lactis *420 at 10^10^ CFU·d^−1^. The *B. lactis *420 supplementation, administered for seven days prior to a 10 mg/kg^−1^ single dose of indomethacin challenge, protected rats from an indomethacin-induced increase in gastric permeability. However, no statistically significant increase due to the NSAID challenge was seen in small intestinal permeability, although the applied dose of indomethacin has previously been shown to be efficient in increasing both gastric and small intestinal permeability [27]. Bifidobacteria have, however, been previously linked with reduced ileal ulceration due to BFMet challenge in rats, with the NSAID-challenged control group displaying ulcerations within the ileum between 18 to 72 hours after NSAID administration [9].

The effect of *B. lactis *420 on the NSAID-induced mucosal damage was not significant, although fewer animals in the probiotic group tended to have the most severe type Grade III lesions in comparison with the NSAID-challenged animals not given *B. lactis *420 (33% versus  50%; Table 2). Possibly the *B. lactis* 420 supplementation protected against more serious GI mucosal damage or enhanced the recovery rate of the stomach mucosa, that is, Grade III lesions were alleviated to Grade II lesions before necropsy. Moreover, the effect of NSAID challenge on TDA values was inconsistent; while in the first trial NSAID induced a clear increase in TDA values, in the second and the third trials, the effect of NSAID was either very small or modest, providing limited scope for improvement.

Although the initial study (a substudy of Study I) had too few rats to show a significant effect, we conducted separate studies for assessing the dose-responsiveness of *B. lactis *420 (four treatment groups, *n* = 40) and the effect of a *B. lactis *420 cell-free extract and lactic acid (five treatment groups, *n* = 74) with the additional gain of elevating the count of animals assayed as in Study I. In the dose-response study, neither of the doses (10^8^ CFU·d^−1^ and 10^10^ CFU·d^−1^) showed a protective effect (Table 4). Due to high variance, the beneficial trend among the average sucrose permeability values was not statistically significant. According to TDA values, the indomethacin challenge was not sufficient in the dose-response study. Nevertheless, for the combined analysis of the control, NSAID and probiotic groups, the data from the dose-response study was also included since the gastric permeability had increased significantly with NSAID administration.

When testing the effect of *B. lactis *420 metabolites and lactic acid, the gastric permeability measurements failed to show adequate NSAID challenge, whereas the lactulose : mannitol-ratio and the TDA values indicated the NSAID challenge to be adequate, but none of the supplementations (live cells, cell-free extract, or lactic acid) were protective. Watanabe and coworkers [19] have previously found a less concentrated (3 to 15 mM) L-lactic acid supplement to protect against mucosal damage in the small intestine of indomethacin-challenged rats. Although speculative, our results indicate the opposite for the effect of 52.4 mM L-lactic acid supplementation on the gastric mucosa. In our study, the concentration of lactic acid was adjusted according to the acetic acid and lactic acid concentrations in the *B. lactis *420 cell-free extract. The results from the metabolite study were also included in the combined analysis.

The three rat studies were conducted in strict accordance with identical timing and dosing procedures to avoid variation. Nevertheless, the effect of the indomethacin challenge on the gastric mucosa varied between both the individual studies and within each study as there was interindividual variation among the rats within each treatment group. In addition to interindividual differences in rat physiology, the subjective visual severity scoring and area estimation applied in the TDA method may have introduced additional variation to the TDA values, although all TDA analyses were performed by the same examiner. The indomethacin batch used in the dose-response study was also different from the one used in the other studies, possibly explaining the low TDA values. A higher indomethacin dose could have reduced the variation in indomethacin-induced GI damage between the studies. However, in a previous study assessing the protective effects of *Lactobacillus casei* strain Shirota, a 10 mg/kg^−1^ single dose was sufficient for gastric ulceration formation in rats [19] and a 25 mg/kg^−1^ already effectively induced gastric ulceration in all rats it was administered to [31]. Moreover, an indomethacin-induced delay in gastric emptying [32] and the rapid healing of the gastric epithelium [31] may hamper the correlation between gastric permeability and TDA measurements.

The sugar permeability values also showed high interindividual variation, but were more comparable between the separate studies. In all rat studies, the animals were given permeability probes 10 hours after indomethacin challenge followed by 15 to 16 hours of urine collection and euthanized alternately from each treatment group starting from 25 hours after indomethacin challenge. Thus the animal study protocol results in an unavoidable, slightly earlier assessment of sugar permeability than the assessment of mucosal damage, which may allow for some degree of healing to occur and therefore affect the TDA results. Kunes et al. also showed that the maximal small intestinal damage is reached in the small intestine of indomethacin-challenged rats only at 48 to 72 hours after indomethacin dosing [31], which is why the TDA of the small intestine was not analysed in this study. Indeed, the protective effect of *B. lactis *420 was significant only regarding the permeability of the stomach, for which the applied protocol time-scale is optimal (Figures 1 and 2(a)). Moreover, the gastric permeability, that is, the amount of sucrose quantified from the rat urine samples, and the presence of mucosal damage in the stomach epithelium did not correlate (see Table 3), although both have previously been reported to increase with indomethacin administration [27].

In a recent study conducted by Senol and colleagues, a probiotic mixture including 13 strains of human origin was effective against aspirin-induced gastric mucosal damage in rats according to macroscopic examination with only ten rats in each treatment group, although the histological analysis showed no effect [20]. In addition to applying a different NSAID than in the present study, their study protocol included both a longer prophylactic probiotic supplementation (14 days) and fasting period prior to the challenge with aspirin (14 hours), while the animals were euthanized just 3 hours after the administration of aspirin. An earlier evaluation of the gastric mucosa could indeed be more efficient in evaluating the gastric mucosal damage as some of the variation may be due to the healing process being already underway and becoming effective. This would, however, hamper the permeability analysis, which was prioritized in our study.

Gotteland and coworkers [13] have successfully applied permeability probes to evaluate the protective effect of a probiotic in a clinical study. They supplemented live and heat-killed LGG cells to 16 human subjects consuming indomethacin. The intestinal permeability was assessed using gastric (sucrose) and small intestinal (lactulose : mannitol) permeability markers which showed a significant protective effect against increased gastric permeability with live LGG cells [13].

Taken together, even though substantial interindividual variation was seen in the manifestation of adverse effects after indomethacin challenge between individual rats, *B. lactis *420 supplementation significantly reduced indomethacin-induced gastric permeability in rats. Based on the results of the present study and previous *in vitro *studies [24–26], testing *B. lactis *B420 in a clinical intervention would be justified.

## Figures and Tables

**Table 1:** Total damaged area of the rat stomach mucosa.

Group^a^	*N*	Mean (mm^2^)	SD	Weighted mean^b^	Weighted SD
Control	40	0.10	0.50	0.12	0.70
NSAID	40	2.74^c^	2.99	3.25^c^	4.43
Probiotic	39	1.91^c^	3.12	2.43^c^	5.00

^a^The treatment groups were control, indomethacin-challenged group (NSAID), and a probiotic group supplemented daily with 10^10^ colony forming units of *Bifidobacterium lactis* 420 for seven days prior to indomethacin challenge.

^b^The weighted values were calculated by multiplying the detected TDA values of increasing severity with 1/10, 3/10, and 6/10, respectively.

^c^ANOVA *P* < 0.0001 when compared with control.

**Table 2:** Prevalence of gastric lesions.

Group/lesion grade^a^	None	Any grade	Grade II or III	Grade III
Control	88	12	5	2
NSAID	20	80^b^	70	50
Probiotic	23	77^b^	67	33

^a^The treatment groups were control, indomethacin-challenged group (NSAID), and a probiotic group supplemented daily with 10^10^ colony forming units of *Bifidobacterium lactis *420 for seven days prior to indomethacin challenge. The lesion Grades I, II, and III represent mild, moderate, and severe lesions, respectively.

^b^ANOVA *P* < 0.05 when compared with control.

**Table 3:** Urinary sucrose levels of rats grouped according to lesion status.

Group^a^	Lesion status	*N*	Urinary sucrose levels	
			Mean (mg/sample)	SD
Control	No lesions	35	16.35	6.72
	Lesions	5	18.86	11.23
	All	40	16.66	7.28
NSAID	No lesions	8	31.56	10.24
	Lesions	32	21.57	9.21
	All	40	23.57	10.13
Probiotic	No lesions	9	17.42	7.64
	Lesions	30	17.45	8.41
	All	39	17.45	8.14

^a^The treatment groups were control, indomethacin-challenged group (NSAID), and a probiotic group supplemented daily with 10^10^ colony forming units of *Bifidobacterium lactis *420 for seven days prior to indomethacin challenge.

**Table 4:** Dose-responsiveness of *Bifidobacterium lactis* 420 against indomethacin-induced side effects.

Group^a^	Quantities of permeability probes^b^ (mg/sample ± SD)		TDA^c^ (mm^2^ ± SD)
	Sucrose	Lactulose : mannitol
Control	12.93 ± 4.08^d^	0.87 ± 0.22	0.00 ± 0.00
NSAID	25.46 ± 8.94^d^	0.85 ± 0.13	3.60 ± 5.13
Probiotic high dose	18.26 ± 7.09	0.79 ± 0.14	1.30 ± 2.68
Probiotic low dose	18.90 ± 6.53	0.87 ± 0.18	2.24 ± 3.15

^a^The treatment groups were control, indomethacin-challenged group (NSAID), and probiotic groups supplemented daily with 10^10^ colony forming units (high dose) or 10^8^ colony forming units (low dose) colony forming units of *Bifidobacterium lactis *420 for seven days prior to indomethacin challenge.

^b^Sucrose measures gastric permeability, and the lactulose : mannitol-ratio reflects small-intestinal permeability.

^c^TDA stands for total damaged area.

^d^Significant (ANOVA; *P* < 0.05) differences between treatment groups are denoted pair wise by superscript letters.

**Table 5:** Effect of *Bifidobacterium lactis* 420 cell-free extract and lactic acid against indomethacin-induced side effects.

Group^a^	Quantities of permeability probes^b^ (mg/sample ± SD)		TDA^c^ (mm^2^ ± SD)
	Sucrose	Lactulose : mannitol
Control	21.63 ± 7.23	1.36 ± 0.28^d, e^	0.70 ± 2.44^f, g, h^
NSAID	28.58 ± 11.38	2.04 ± 0.45^d^	6.28 ± 6.85^f^
Live cells	19.71 ± 10.61	1.83 ± 0.53^e^	2.84 ± 3.12^g^
Cell free extract	22.48 ± 14.41	1.60 ± 0.51	1.88 ± 2.41
Lactic acid	24.90 ± 12.24	1.76 ± 0.38	7.48 ± 9.21^h^

^a^The treatment groups were a control group, an indomethacin-challenged group (NSAID), and groups supplemented daily with 10^10^ colony forming units of *Bifidobacterium lactis *B420 (live cells), *B. lactis *420 cell-free extract (cell-free extract), or lactic acid for seven days prior to indomethacin challenge.

^b^Sucrose measures gastric permeability and the Lactulose : mannitol-ratio reflects small-intestinal permeability.

^c^TDA stands for total damaged area.

^d-h^Significant (ANOVA; *P* < 0.05) differences between treatment groups are denoted pair wise by superscript letters.
